# Carbamazepine-Induced Toxic Epidermal Necrolysis Managed by Mobile Teledermatology in COVID-19 Pandemic in Rural Nepal

**DOI:** 10.1155/2020/8845759

**Published:** 2020-11-06

**Authors:** Vikash Paudel, Deepa Chudal

**Affiliations:** ^1^National Medical College, Birgunj, Parsa, Nepal; ^2^Nepal Police Hospital, Kathmandu, Nepal

## Abstract

Toxic epidermal necrolysis is a life-threatening dermatological emergency with high mortality if not treated in time. Here we report a case of toxic epidermal necrolysis due to carbamazepine in rural Nepal in COVID-19 pandemic who was successfully treated with the help of mobile teledermatology. The clinical impression of toxic epidermal necrolysis was made from “WhatsApp” video calls using a smart phone. The supportive features were the history of starting of carbamazepine 2 weeks prior for seizure disorder, clinical findings in serial photographs of skin with 40 percent body surface area involvement of necrotic skin lesions and bulla, and involvement of oral mucosa and eyes. The patient was immediately asked to stop carbamazepine and was treated with intravenous fluids and systemic steroids along with symptomatic management. As the whole world was suffering from lockdown due to COVID-19 crisis, it was impossible for the rural area patient to visit a dermatologist. Thus, with the help of paramedics staff in a community health center and mobile teledermatology, the patient was diagnosed as carbamazepine-induced toxic epidermal necrolysis and treated successfully with good outcome.

## 1. Introduction

Toxic epidermal necrolysis (TEN), also known as Lyell's syndrome [1], is a potentially life-threatening dermatological emergency with high mortality [2]. Though TEN is less common in dermatological practice, carbamazepine is one of the commonest culprits among all the drugs [3]. Here, we present a case of carbamazepine induced toxic epidermal necrolysis in a young female from rural Nepal, who was successfully treated using simple mobile teledermatology in COVID-19 pandemic.

## 2. Case Report

This is a story of a 12-year-old female from rural Nepal who developed rapidly progressive generalized rash along with mild fever and headache. She was diagnosed with seizure disorder 2 weeks prior to the current illness and was prescribed carbamazepine at the dose of 200 mg daily. On the 14^th^ day of carbamazepine, she developed those rashes which were purpuric, some of which later turned to blisters, dusky colored, itchy to start with which later were tender, and covered almost 40% of body surface area covering trunk and face with involvement of oral mucosa and eye. It was associated with swelling of the face and mild fever.

As the whole country was under lockdown due to COVID-19 pandemic, it was impossible for the rural patient to visit the dermatologist. For this, she went to a nearby community health center, from where mobile teleconsultation was made with the help of a community health worker (health assistant).

**Figure 1:**
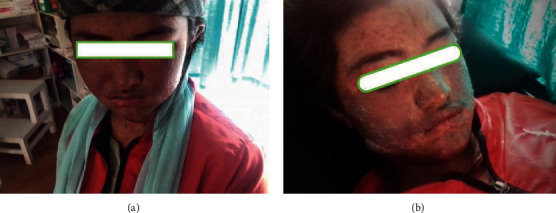
(a, b) Erythema and vesicobullous lesions involving the face and neck.

**Figure 2:**
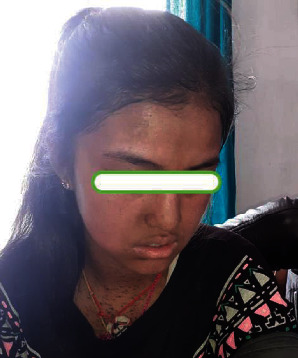
Patient during discharge from the health center at 2 weeks.

On assisted physical examination with the help of the health worker, it was found that the patient was febrile with areas of blistering and peeling of the skin involving face, trunk, and extremities with crusting seen over lips and eyes (Figures 1 and 2). The Nikolsky sign was positive. The erythematous rash was covered almost all over the trunk with epidermal detachment of 40% body surface area.

The clinical impression of toxic epidermal necrolysis due to carbamazepine was made after taking detail history and serial clinical findings in video calls and photos sent in the mobile device.

**Figure 3:**
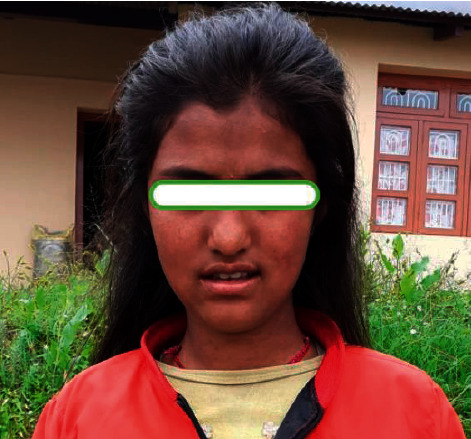
Patient on follow-up at 4 weeks.

Regarding the management of the patient in rural setting, carbamazepine was stopped immediately. She was supplemented with a rapidly tapering dose of intravenous hydrocortisone along with fexofenadine, mupirocin ointment, and moisturizer. The erosions were smeared with mupirocin and paraffin gauges. Supportive treatment given included parenteral analgesics for pain management and intravenous fluids. The patient was advised for better nutrition for healing of the wound by giving adequate carbohydrate, high-protein diet, and vitamin supplements. She was switched over to oral prednisolone once the lesions started healing after five days. Later on, prednisolone was slowly tapered slowly over two weeks. The lesions healed with postinflammatory hyperpigmentation (Figures 2 and 3).

## 3. Discussion

TEN is a severe cutaneous adverse drug reaction and life-threatening dermatological emergency [1]. The common causes are mostly drugs like sulphonamides, nonsteroidal anti-inflammatory drugs (NSAIDs), beta-lactum antibiotics, quinolones, nevirapine, antitubercular drugs, allopurinol, and aromatic antiepileptics like carbamazepine, phenobarbital, and phenytoin [3]. The time duration of TEN after initiating carbamazepine is usually less than 3 weeks. Our patient presented with generalized erythema and peeling of the skin with mucosal erosions two weeks after initiating carbamazepine.

Recently, carbamazepine was found to be a common drug causing TEN [4]. The pathogenesis of TEN is cytotoxic destruction and apoptosis of keratinocytes. The proapoptotic molecules like TNF-*α*, interferon-*γ*, and inducible nitric oxide synthase may link drug-induced immune responses to keratinocyte damage [3, 5]. Soluble Fas ligands, perforin, granzyme, and, recently, granulysin have been implicated in death of keratinocyte [6]. Drug metabolites also act as haptens, and defect in the detoxification system of clearing those haptens may be the cause of severe drug reactions [3].

As the patient was from a very rural area without proper healthcare, even without a medical doctor, the access to dermatologist was unimaginable. However, the mobile teledermatology assisted with proper diagnosis and management of the case. The foremost part in the management of such severe drug reaction was to stop the culprit drug. The specific treatment modalities in management are high dose of corticosteroid (though debated), cyclosporine, plasmapheresis, and intravenous immunoglobulins [7]. We abruptly stopped carbamazepine. As the patient had only access to systemic steroid and thus was advised to administer intravenous hydrocortisone which was later switched to oral prednisolone.

Teledermatology is increasingly being used worldwide in these pandemics situations than ever, and mobile teledermatology is its simple form. This could also be a dynamic tool for the remote areas where a dermatologist is not available, even in post-COVID era as well [8]. They could be life-saving, timesaving, and economical at times like our scenario [9].

Carbamazepine-induced TEN was successfully managed at the outreach center with the help of mobile teledermatology. Awareness about the drugs implicated in severe drug reactions would help to prevent these life-threatening drug reactions. Besides, the use of teledermatology would help the healthcare workers and medical officers with less exposure of dermatology in identifying and managing skin diseases.
